# Quantifying the economic value of earlier and enhanced management of anorexia nervosa for adults in England, Germany and Spain: improving the care pathway

**DOI:** 10.1192/j.eurpsy.2024.1751

**Published:** 2024-05-23

**Authors:** David McDaid, Janet Treasure, Fernando Fernández-Aranda, Beate Herpertz-Dahlmann, Vinciane Quoidbach, Suzanne Dickson, Philip Gorwood

**Affiliations:** 1 Care Policy and Evaluation Centre, Department of Health Policy, London School of Economics and Political Science, London, UK; 2 Centre for Research in Eating and Weight Disorders (CREW), Institute of Psychiatry, Psychology and Neuroscience, King’s College London, London, UK; 3 Psychoneurobiology of Eating and Addictive Behaviours Group, Neurosciences Programme, Bellvitge Biomedical Research Institute (IDIBELL), Barcelona, Spain; 4 Department of Clinical Psychology, Bellvitge University Hospital, Barcelona, Spain; 5 CIBER Fisiopatología Obesidad y Nutrición (CIBERObn), Instituto de Salud Carlos III, Madrid, Spain; 6 Department of Clinical Sciences, School of Medicine and Health Sciences, University of Barcelona, Barcelona, Spain; 7 Department of Child and Adolescent Psychiatry, Psychosomatics and Psychotherapy RWTH Aachen University, Aachen, Germany; 8 European Brain Council, Brussels, Belgium; 9 Institute of Neuroscience and Physiology, The Sahlgrenska Academy at the University of Gothenburg, Gothenburg, Sweden; 10 Université Paris Cité, GHU ParisPsychiatrie et Neurosciences, CMME, Paris, France; 11 INSERM U1266, Institute of Psychiatry and Neurosciences of Paris (IPNP), Paris, France

**Keywords:** anorexia nervosa, economic modelling, enhanced care pathways, healthcare costs, net monetary benefits

## Abstract

**Background:**

Anorexia nervosa (AN) is a serious mental illness. One-third of people develop severe, enduring, illness, adversely impacting quality of life with high health system costs. This study assessed the economic case for enhanced care for adults newly diagnosed with AN.

**Methods:**

A five-state 312-month-cycle Markov model assessed the economic impact of four enhanced care pathways for adults newly diagnosed with AN in England, Germany, and Spain. Enhancements were halving wait times for any outpatient care, receiving specialist outpatient treatment post-referral, additional transitional support post-referral, and all enhancements combined. Care pathways, estimates of impact, resource use, and costs were drawn from literature. Net monetary benefits (NMBs), impacts on health system costs, and disability-adjusted life years (DALYs) averted were estimated. Parameter uncertainty was addressed in multi-way sensitivity analyses. Costs are presented in 2020 purchasing power parity adjusted Euros.

**Results:**

All four enhanced care pathways were superior to usual care, with the combined intervention scenario having the greatest NMBs of €248,575, €259,909, and €258,167 per adult in England, Germany, and Spain, respectively. This represented maximum NMB gains of 9.38% (€21,316), 4.3% (€10,722), and 4.66% (€11,491) in England, Germany and Spain compared to current care. Healthcare costs would reduce by more than 50%.

**Conclusions:**

Early and effective treatment can change the trajectory of AN. Reducing the untreated duration of the disorder is crucial. There is a good economic case in different country contexts for measures to reduce waiting times between diagnosis and treatment and increase access to enhanced outpatient treatment.

## Introduction

Anorexia nervosa (AN) is a serious mental illness [1] with typical onset in adolescence and a protracted course. Over one-third of people develop severe and enduring illness (SE-AN) [2–4]. Lifetime prevalence is estimated at 2–4% among women and 0.3% among men [5]. A total of 153,058 disability-adjusted life years (DALYs) were due to AN in the WHO European Region in 2019, 78% for women [6]. The long duration of illness means that 117,946 (77%) of DALYs are for people aged over 20.

Although incident rates for AN peak in early adolescence, they remain high for young women, in particular; for example, Swedish registry data indicate 149, 95, and 40 AN cases per 100,000 women aged 18–19, 20–23, and 24–30; for men, these rates are 3.3, 2.9, and 1.0 [7]. The COVID-19 pandemic exacerbated the challenge. Systematic reviews, surveys, and record studies with evidence from England, France, Germany, Ireland, Netherlands, Spain and Sweden suggest increased hospitalisation and AN diagnosis during the pandemic [8–12]. Analysis of 9 million English primary care records reported an increase in eating disorder (ED) incidence in women aged 17–19 (32%) and 20–24 (14%) between 2020 and 2022 [13].

AN can have profound consequences. Malnutrition contributes to a wide range of physical and psychological disabilities which can severely disrupt physical, cognitive, socio-emotional and educational development. Metabolically active organs, such as the brain, are particularly impacted with acute AN having a bigger effect on brain structure than other mental health conditions. For example, a 6% reduction in size of brain cortex has been shown [14]. Numerous psychological features include problems in cognitive flexibility [15], memory [16] and social cognition [17]. A meta-analysis estimated prevalence of suicidal intentional self-harm at 17% among people with AN [18], while all-cause mortality rates are the highest of any mental illness [19].

Specific personality traits and psychological comorbidities, such as mood and anxiety disorders, are common, contributing to adverse outcomes [20]. People with co-morbid depression are six times more likely to remain unrecovered after 22 years compared to those without depression [2]. Enduring illness has been associated with cognitive, behavioural and neurobiological changes, adversely impacting treatment outcomes [21–23].

Healthcare costs associated with AN are high; costs of failing to treat effectively and early are numerous [19, 24]. Average admission length in Europe is 106 days [25]; readmissions may be even longer [26]. In the UK, AN inpatient admissions have increased annually over the last two decades [27]. Evidence on educational attainment is equivocal; longitudinal studies in Norway and Sweden find little impact of EDs [28, 29], but studies indicate AN can lead to reduced workforce participation, higher absenteeism/presenteeism and lower earnings when employed [30].

A systematic review reported AN was associated with reduced mobility compared to bulimia nervosa and healthy controls [31]. The illness also has considerable negative impact not only on patients’ health and wellbeing, but also on their immediate environment, posing substantial challenges to primary caregivers and families [32].

Guidelines on management of AN are available internationally, for instance in England, they recommend outpatient psychotherapy, which can lead to good outcomes, especially when accessed early [33]. However, despite adverse health and economic consequences, evidence on the extent and quality of guideline implementation is limited. Challenges include availability of specialist treatment, as well as the lack of resources, including knowledge of ED in primary care, beds and trained therapists. Reviews, mainly of European studies, indicate average duration of untreated AN between 15 months and 2 years [22, 34], with long periods of time between disorder onset, diagnosis, assessment and commencement of treatment [22, 35]. Delays in accessing treatment may be partly due to individuals not seeking help, as it is often the concern of others (e.g., parents) that brings them to treatment. Many people with AN, therefore, still receive no ED-specific treatment and/or experience delays in treatment, while some remain completely untreated [34, 36].

Even when treated, a large proportion of individuals with SE-AN fail to respond to outpatient treatment; 20–30% may require rescue treatment, such as inpatient or day patient care, of which, 30–40% require repeated readmissions [20, 37]. Earlier and easier access to specialist services can prevent a protracted course of illness and improve outcomes [38]. A new form of early intervention the First Episode Rapid Early Intervention for Eating Disorders (FREED) for young adults (aged 16–25) in England has been able to shorten some service-related delays, with potential for improving outcomes [39] and reducing costs [40].

There is some further limited economic evidence base on treatments for AN in adults; a recent systematic review [41] identified a German analysis where focal psychodynamic therapy and cognitive behavioural therapy (CBT) had better outcomes and lower costs than care as usual for women [42], while high calorie refeeding was associated with lower hospital costs in a U.S. trial [43]. In a pre–post study in the Netherlands, CBT had higher costs per remission gained but it is unclear whether this is cost effective [44]. Other than FREED, no other economic evaluations looking at the benefits of reduced wait times and/or earlier access to specialist care pathways were identified.

Given this context, this study is a follow-up to European Brain Council (EBC) initiatives to estimate the burden and costs associated with disorders of the brain in Europe in 2010, which found that people with ED incurred the highest proportion of direct healthcare costs (72%) [45]. In 2015, the EBC initiated the value of treatment (VOT) research framework to investigate unmet needs in healthcare and the increasing all-age burden of brain disorders (both neurological and mental). A second round (VOT2) on new therapeutic areas (AN, autism spectrum disorder and major depressive disorder) launched in 2019 and produced a review of care pathways for adults with AN [46]. These pathways might benefit from improvements to transition points into care, or between levels and stages of care. Potentially, improvements, including early access to treatment, availability of effective treatments, and support for transitions out of tertiary services, might also be cost-effective. The aim of this study, therefore, was to model different enhanced care pathway scenarios showing their potential health and economic impacts in England, Germany and Spain.

## Methods

Health economic modelling studies are widely used to help determine the potential strength of investment in different options for better health and wellbeing [47]. Models bring together evidence on effectiveness, resource use and costs from multiple sources. One approach is Markov modelling. It can be used to model uncertain processes over multiple time periods known as cycles and reflect circumstances, as for AN, where individual health outcomes can fluctuate [48].

A five-state Markov model was constructed to compare five potential care pathways for an adult with newly diagnosed AN in England, Germany, and Spain. The model was developed using TreeAge Pro Healthcare 2023 [49] and runs over 312 weeks (6 years) with each Markov cycle lasting 1 week, comparing typical wait times and then subsequent use of outpatient and inpatient ED treatment after AN diagnosis.

**Figure 1.**
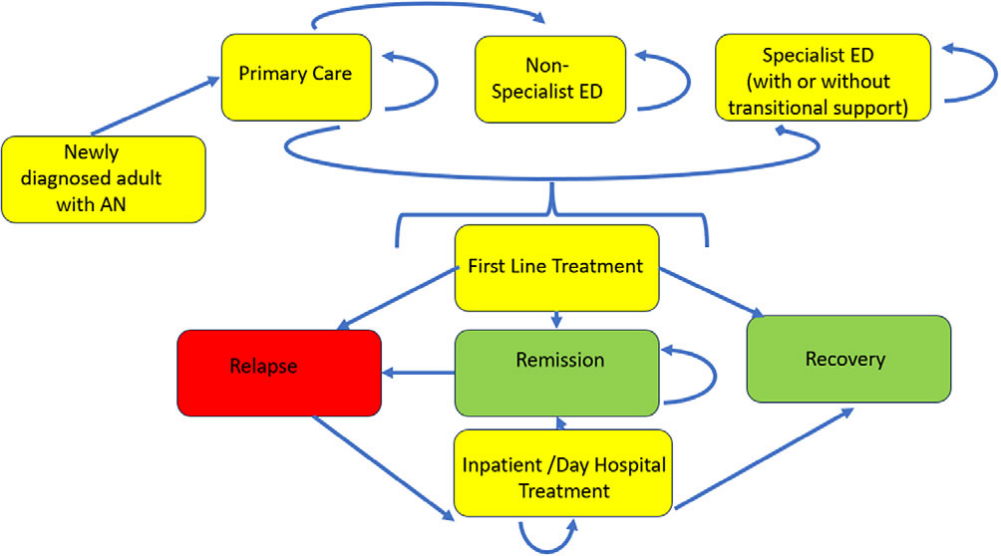
State transition diagram. A newly diagnosed individual may just receive one of the front-line treatments or a combination of treatments upon entry into the mode. The amount of time spent in remission before relapse can vary and includes the possibility of immediate relapse and immediate hospital treatment after the completion of outpatient treatment.

**Figure 2.**
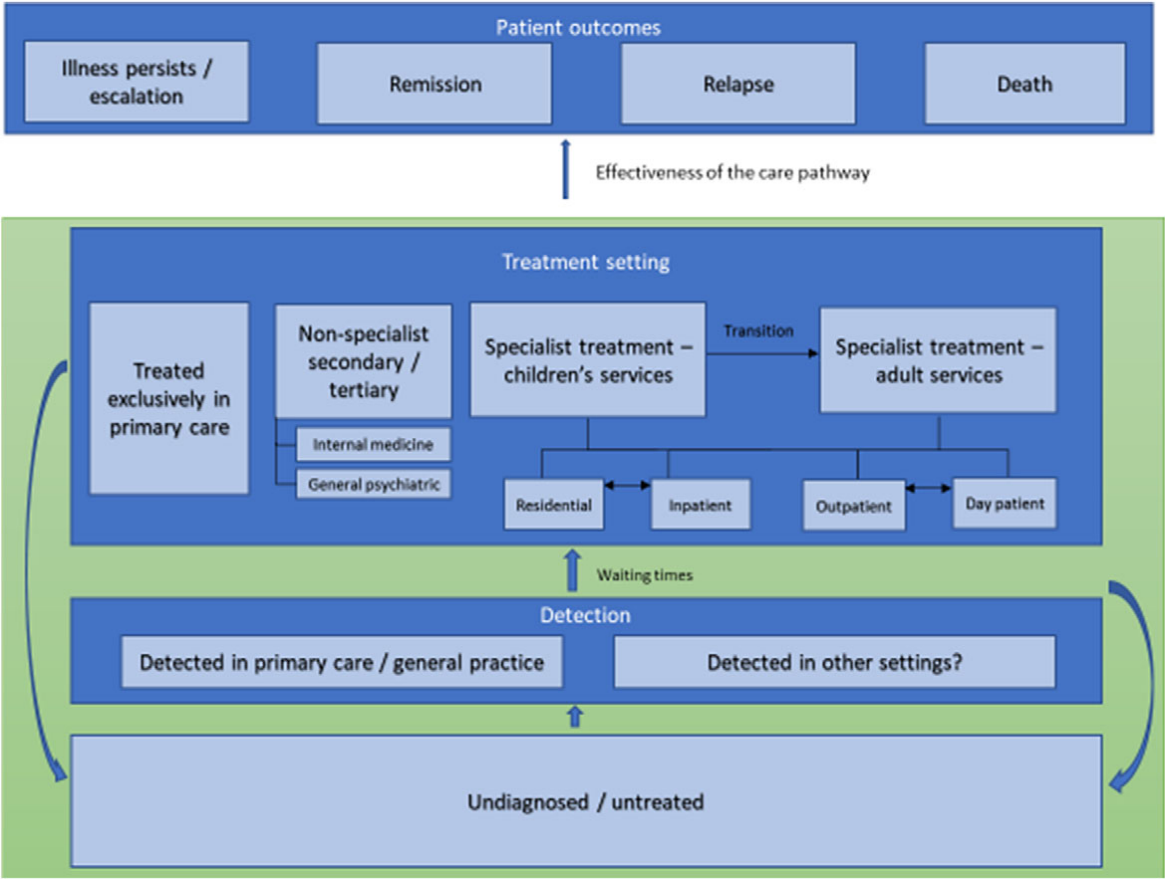
Schematic care pathway for anorexia nervosa in Europe.

Figure 1 provides an overview of model health states. Figure 2 provides a schematic for AN care. Potential changes to enhance transition points post-diagnosis on this care pathway to model were drawn from the EBC’s previous review [46].

### Care pathway scenarios

In our model, individuals enter when initially diagnosed with AN. *Scenario 1*, the baseline scenario, is a current care pathway based on existing data on waiting times, hospitalisation rates, length of inpatient stays and rehospitalisation rates, as well as current best practice recommendations for AN treatment [25]. It assumes people with AN are monitored in primary care, with no waiting period prior to accessing primary care. After this watchful waiting period, individuals may be treated in outpatient specialist ED services or non-specialist services. In line with current English National Institute for Health and Care Excellence (NICE) recommendations for adults, we assume specialist delivered care is either the Maudsley anorexia treatment for adults (MANTRA) or specialist supportive clinical management (SSCM) [33].

Treatment is assumed to last 20 weeks; the model assumes in each subsequent weekly cycle, there are three possible states: recovery, remission, or relapse requiring a period of hospitalisation within 2 years, with the possibility of a further period of rehospitalisation over an additional 1-year period. This includes the possibility of immediate relapse, recognising the risk of immediate failure of initial treatment.

Four enhanced care pathways are considered. *Scenario 2* looks at potential impacts of halving mean waiting times for outpatient treatment. Reduced wait time may be associated with better outcomes. Early interventions may also improve outcomes, as seen for example in the adult Spanish ED population, especially for those with subthreshold ED [20]. As the initial rate of hospitalisation following treatment in specialist ED services is lower than for non-specialist treatment, *Scenario 3* examines the impact of providing specialist treatment for everyone following referral. *Scenario 4* includes additional transition support, such as a hypothetical carer-focused intervention for those receiving specialist ED care. This is assumed to further reduce the rate of relapse and hospitalisation for those receiving specialist ED care by 50% compared to receipt of specialist ED care alone. *Scenario 5* combines all three enhancements to the care pathway.

The primary outcome is DALYs averted. A disability weight of 0.224 [50] was assigned to all time spent in states waiting, or receiving treatment, for AN. This is conservative, as it assumes that no DALYS were averted during periods of treatment. Recovery and remission states were assumed to incur no disability. The maximum possible DALYs averted per individual would be 6 (or 5.43 when discounted), if all time over the 6-year period was spent AN free.

Mean costs associated with AN events in each cycle were computed. All costs are presented in 2020 purchasing power parity adjusted (PPP) Euros using values (including UK) from Eurostat [51]. Where necessary, raw costs were first adjusted to 2020 prices using country-specific GDP deflators [52, 53]. An annual discount rate of 3.5% was applied to outcomes and costs. The economic analysis was undertaken from a health and social care system perspective.

In addition to estimating expected costs and DALYs averted for each scenario, net monetary benefits (NMBs) associated with each model scenario were calculated using a notional willingness to pay threshold of €50,000 per DALY averted. NMB allows for transparent comparison of multiple strategies, including variation of willingness to pay thresholds, and can be used to rank different care pathway scenarios. Sensitivity analyses were performed varying all key parameters to see what impact this had on care pathway scenario ranking and magnitude of economic benefits gained. A CHEERS (Consolidated Health Economic Evaluation Reporting Standards) checklist is included in the Supplementary Material [54].

## Model parameters

Table 1 provides an overview of parameters used, including country-specific unit costs for health services, as well as distributional assumptions. Country-specific estimates of time-waiting before treatment were obtained. In England, average duration of waiting time from first primary visit to referral and then treatment in a mixed population was reported at 27 weeks for people aged 19 and over [55]. A later study for people aged 16–25 with an ED, 52% of whom had AN, also reported a mean 27 weeks just for the period from referral to treatment [56]. In Germany, average duration of wait time between disorder onset and treatment for AN is 12 months [57], while in Spain average waiting time from onset of AN to first contact with services is 13.05 months [58]. Adjusting these latter two wait times to reflect the wait time period between primary care referral and treatment in England, where 85% of total waiting time fell between onset and primary care referral, average waiting times in Germany and Spain would be 7.8 and 8.775 weeks, respectively. Our base case scenario conservatively assumed a high proportion of people (70%) would be treated in specialist services in all three countries, in line with previous estimates for young adults [59, 60].

**Table 1.** Model parameters (all costs in 2020 PPP adjusted Euros)

Input parameter	Deterministic value	Distribution	Source
Waiting time			
Mean time from help–seeking to treatment (mixed ED population) (England) (weeks)	27	Normal	[55, 56]
Mean time from help–seeking to treatment for anorexia nervosa (Germany) (weeks)	7.8	Normal	[57]
Mean time from help–seeking to treatment for anorexia nervosa (Spain) (weeks)	8.775	Normal	[58]
DALY weights			
Remission/recovery from eating disorder	0	Beta	[50]
Living with anorexia nervosa	0.224	Beta	[50]
Health service unit costs (England)			
Adult specialist ED services, admitted patient (per day)	€686.03	Gamma	[86]
Non–specialist outpatient care (per contact)	€198.56	Gamma	[86]
Adult specialist ED service, outpatient care (per contact)	€277.74	Gamma	[86]
GP consultation (per contact)	€42.80	Gamma	[87]
Health service unit costs (Germany)			
Adult specialist ED services, admitted patient (per day)	€388.77	Gamma	[88]
Non–specialist outpatient care (per contact)	€42.57	Gamma	[88]
Adult specialist ED service, outpatient care (per contact)	€89.36	Gamma	[88]
GP consultation (per contact)	€22.96	Gamma	[88]
Health service unit costs (Spain)			
Adult specialist ED services, admitted patient (per day)	€454.66	Gamma	[89]
Non–specialist outpatient care (per contact)	€75.13	Gamma	[89]
Adult specialist ED service, outpatient care (per contact)	€121.54	Gamma	[90]
GP consultation (per contact)	€26.11	Gamma	[89]
Specialist day care	€105.21	Gamma	[90]
Length of hospital stay (weeks)			
England: inpatient	16.00	Normal	[25]
Germany: inpatient	13.42	Normal	[25]
Spain: inpatient	10.71	Normal	[25]
Spain: day hospital following inpatient stay	15.00	Normal	[20, 63, 64]
Other probabilities			
Probability of being treated with specialist ED outpatient/day care	0.7	Beta	[59, 60]
Probability of being treated with non–specialist ED outpatient/day care	0.3	Beta	[59, 60]
Probability of hospitalisation following specialist ED treatment	0.17	Beta	[61, 62]
Probability of hospitalisation following non–specialist ED treatment	0.40	Beta	[61, 62]
Probability of rehospitalisation	0.412	Beta	[20]
Maximum length of time to rehospitalisation (weeks)	52	Normal	[37]
Discount rate (after 12 months)	0.035	Fixed	[91]

Likelihood of hospitalisation in all countries following non-specialist care was assumed to be 40%, compared with 17% for those who received specialist care, based on experience with SSM and MANTRA [61, 62]. The rate of rehospitalisation was conservatively assumed at 41.2% in all three countries based on longitudinal data of adults with AN in Spain [20]. The model assumes re-hospitalisation occurs within 12 months of discharge from initial hospitalisation, in line with previous analysis [37].

Length of inpatient stay was drawn from a recent review [25]. Country-specific values were calculated as a weighted average. As only one study was from Spain, all calculations also include two studies which drew on European populations. Average length of stay was 16 weeks for England, 13.42 weeks for Germany, and 10.71 weeks for Spain. In Spain, shorter inpatient admission is usually followed by a lengthy day-hospital stay, this averaged at 15 weeks [20, 63, 64] and was included in the Spanish model.

## Results

Tables 2–4 show the costs of each of the five scenarios, DALYs averted and NMB in each country. The potential economic case is greatest for the Scenario 5 strategy that both substantially reduces wait times for contact with outpatient services, as well as increasing access to enhanced specialist care. The potential maximum NMBs are €248,575, €259,909 and €258,167, respectively, in England, Germany, and Spain, with gains of 9.38%, 4.30% and 4.66% compared to current care pathways. Scenario 4 which adds further transitional support for people receiving outpatient specialist care has the second-most NMB in all countries. Scenario 2 where waiting times for treatment are halved is the third ranked scenario in England and Germany, while Scenario 3 which ensures all people with AN receive specialist outpatient care is third ranked in Spain.

**Table 2.** Expected costs, DALYs averted and net monetary benefits for each anorexia nervosa care pathway – England (€’s 2020 PPP adjusted)

Costs (€s)	Current	Halving wait times	Specialist access for all	Additional transitional support	Combination
Primary care management	1,315	672	1,315	1,315	672
Non–specialist outpatient care	1,217	1,229	0	1,217	0
Specialist outpatient care	3,972	4,013	5,675	3,972	5,732
Inpatient care	25,240	21,220	17,953	16,085	7,991
Total cost	31,744	27,134	24,943	22,589	14,395
DALYs					
DALYs averted	5.181	5.248	5.187	5.188	5.259
Incremental DALYs averted versus current care pathway		0.067	0.006	0.007	0.078
Net monetary benefits (NMBs) (€s)	227,259	235,243	234,387	236,824	248,575
NMB gain versus current care pathway (€s)		7,984	7,128	9,565	21,316
NMB gain versus current care pathway (%)		3.51%	3.14%	4.21%	9.38%

**Table 3.** Expected costs, DALYs averted and net monetary benefits for each anorexia nervosa care pathway – Germany (€’s 2020 PPP adjusted)

Costs (€s)	Current	Halving wait times	Specialist access for all	Additional transitional support	Combination
Primary care management	335	82	335	335	82
Non–specialist outpatient care	260	266	0	260	0
Specialist outpatient care	1,271	1,301	1,816	1,271	1,859
Inpatient care	11,655	9,976	8,290	7,428	3,757
Total cost	13,521	11,625	10,441	9,294	5,698
DALYs averted	5.254	5.302	5.259	5.261	5.312
Incremental DALYs averted versus current care pathway		0.048	0.005	0.007	0.058
Net monetary benefits (NMBs) (€s)	249,187	253,489	252,533	253,748	259,909
NMB gain versus current care pathway (€s)		4,302	3,346	4,561	10,722
NMB gain versus current care pathway (%)		1.73%	1.34%	1.83%	4.30%

**Table 4.** Expected costs, DALYs averted and net monetary benefits for each anorexia nervosa care pathway – Spain (€’s 2020 PPP adjusted)

Costs (€s)	Current	Halving wait times	Specialist access for all	Additional transitional support	Combination
Primary care management	256	104	256	256	104
Non–specialist outpatient care	467	469	0	467	0
Specialist outpatient care	1,763	1,770	2,518	1,763	2,528
Day hospital care	1,871	1,879	1,331	1,433	707
Inpatient care	11,969	10,011	8,514	7,636	3,770
Total cost	16,326	14,233	12,619	11,555	7,109
DALYs averted	5.261	5.287	5.269	5.269	5.306
Incremental DALYs averted versus current care pathway		0.026	0.008	0.008	0.045
Net monetary benefits (NMBs) (€s)	246,676	250,142	250,838	251,920	258,167
NMB gain versus current care pathway (€s)		3,466	4,162	5,244	11,491
NMB gain versus current care pathway (%)		1.41%	1.69%	2.13%	4.66%

**Figure 3.**
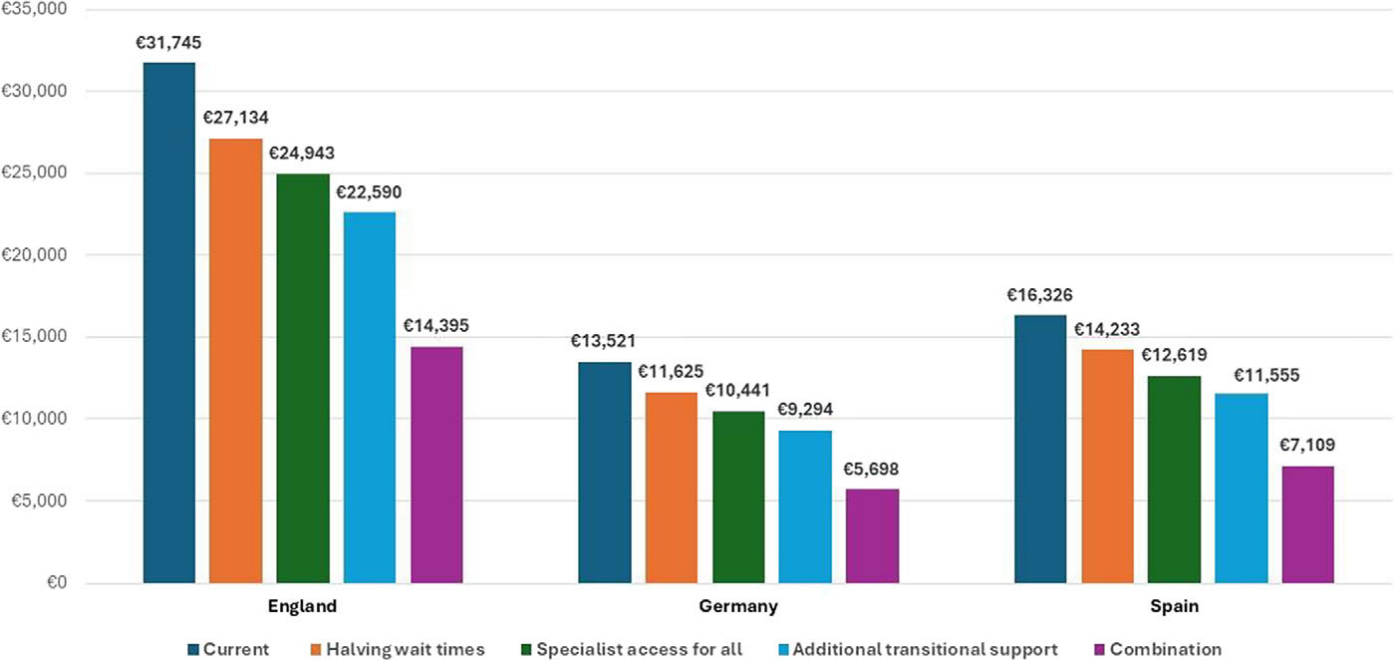
Expected mean 6-year costs of anorexia nervosa care pathways per country and scenario (2020 PPP adjusted Euros).

**Figure 4.**
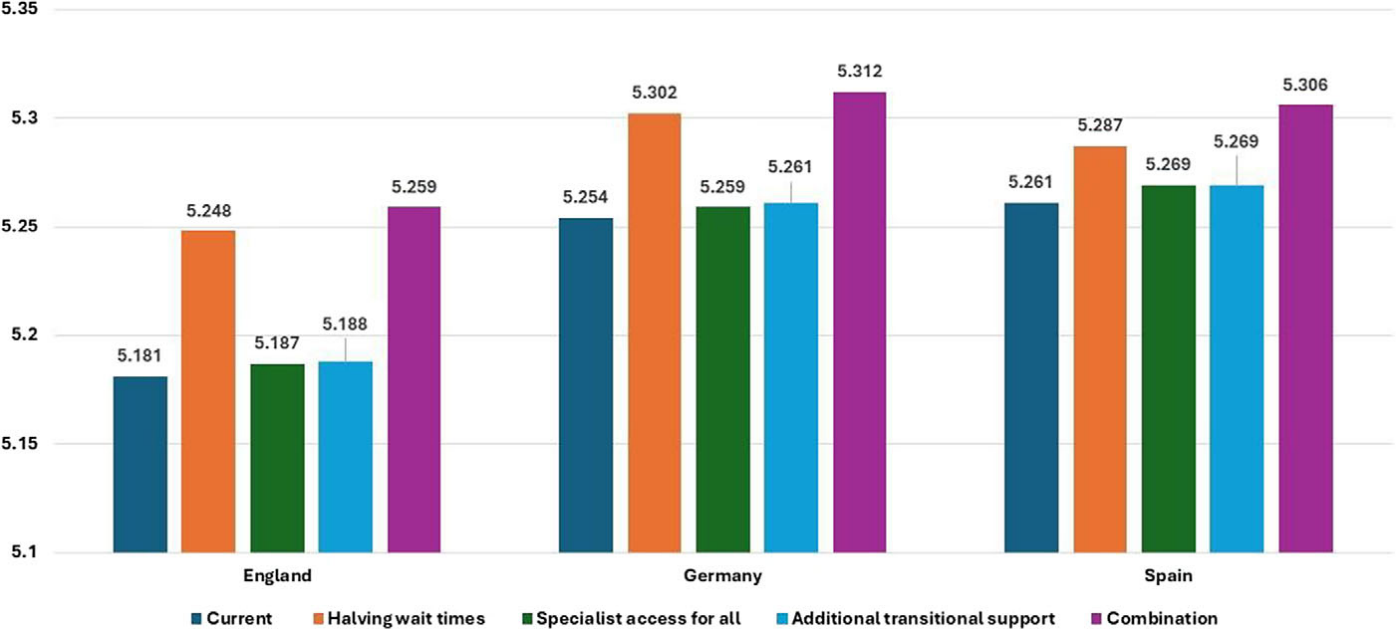
Expected mean disability-adjusted life years (DALYs) averted of care pathways per country and scenario.

Figures 3 and 4 show total expected costs and expected total DALYs averted per person with AN for each care pathway scenario in each country. In Figure 3, costs are consistently highest in the baseline Scenario 1 and consistently lower in each subsequent scenario. The reductions in expected care pathway treatment costs between Scenarios 1 and 5 in England, Germany and Spain are 54.65%, 57.86% and 56.46%, respectively. Increased access to specialist services, and thus reduced risk of further hospitalisations, drives these cost reductions. Figure 4 indicates the key driver of increasing the number of DALYs averted in all countries is reducing length of time waiting for treatment. Gains are greatest in England due to longer base case wait times. In all cases, DALYs averted are maximised in Scenario 5.

### Sensitivity analyses

One-way sensitivity analyses were conducted to look at how changes in model parameters impact on expected NMB and relative ranking of care pathway scenarios. Key parameters were varied 20% above/below baseline values. The DALY disability weight for AN was varied between its 95% confidence intervals, while the disability weight for remission/recovery was varied between 0 and the lower 95% confidence interval for living with AN (0.15). Scenario 2 already indicated the model is sensitive to duration of expected wait time prior to access to specialist outpatient services; the longer the wait time, the greater the economic case for action, however all other parameters, including length of hospitalisation, specialist versus non-specialist outpatient care services and inpatient care costs have little impact on model results and ranking of scenario NMBs (see Supplementary Material). We also undertook probabilistic sensitivity analysis varying key parameters concurrently 10,000 times. Again, this did not change scenario rankings or magnitude of NMBs (see Supplementary Material).

## Discussion

This study aimed to estimate the value of investing in enhanced care pathways for management of AN for adults in England, Germany and Spain. The model demonstrates that an enhanced care pathway strategy combining measures to reduce waiting time for specialist care, as well as use of specialist rather than non-specialist outpatient ED services, supplemented by additional transitional support, such as carer-focused interventions, generates the highest levels of NMB. This reflects both lower health system costs and higher levels of DALYs averted.

These results are in line with research indicating early and effective treatment can change the trajectory of AN and prevent it from becoming protracted. A German randomised controlled trial of psychotherapy in outpatients with AN followed-up over 5 years showed earlier treatment in the course of the illness achieved better long-term outcomes [65]. Although a recent review indicated there are few economic analyses looking at treatment of AN in adults [41], there is some prior economic evidence for early intervention and reduced wait time for adults. A quasi-experimental evaluation of the FREED model of early intervention in England indicated the chance of reaching a healthy weight at 12 months follow-up was tripled, with no statistically significant difference in costs between FREED and care as usual groups [40]. Modelling analyses in Germany also indicate a positive economic case for expanding access to psychological treatment in adults [66].

While our modelling suggests a good economic case for enhancing care pathways, this raises significant policy, resource and implementation challenges. While the resource savings as a result of reduced inpatient stays are substantial, we have not made any assumptions about the approach used to reducing wait times; this will not be costless. Approaches could include regulatory measures, such as waiting time targets; for example, these exist in England, but need more substantial monitoring to be effective [67]. There also needs to be investment in measures to achieve greater awareness among primary care practitioners of the importance of early intervention and more rapid access to specialist support [68,69]. If wait times are to be cut, there also needs to be investment in supply-side measures to increase capacity in outpatient care. In Germany, for example, numbers of qualified psychotherapists and psychiatrists experienced in ED to provide outpatient treatment are insufficient, even though there are sufficient inpatient and day patient beds. Without commitment to upfront investment for more psychotherapists in Germany, there may be pressures to instead rely more on existing, but more expensive, inpatient care. Thus, resource requirements and costs associated with scaling-up the workforce, as well as raising awareness in primary care practitioners and enforcement of wait time targets need to be considered in future modelling analyses.

Reducing waiting times may also impact on the chance of developing SE-AN, especially in sub-threshold AN cases [20] and reduce mortality risk [4]. While greatest benefits are gained from increased access to specialist outpatient care, our model indicates any measures that increase access to appropriate non-specialist outpatient care are of benefit. Improved training and support may be of value for these broader outpatient services, given the likely time-lag in expanding access to more specialist services. This is recognised in England, where Health Education England has expanded training for outpatient teams and specialist groups in MANTRA and CBT for ED [70].

Inpatient stays are a large driver of costs in ED. Our model does not consider outpatient or home-treatment interventions that reduce hospitalisation. These have promise and may reduce costs, although more support may be needed from family carers [71, 72]. Evaluation in a large-scale trial in Germany is underway [73]. Interventions such as skills training for caregivers (Experienced Caregivers Helping Others, ECHO), as well as other online and transition supports that help sustain effects of outpatient treatments should also be prioritised, in addition to development of highly effective first-line treatments [74, 75]. Digital approaches that are highly accessible and scalable may also offer opportunities for improved outcomes and greater cost savings.

Our model indicates a substantial economic case for care pathway enhancement, yet our estimates of benefits are likely to be conservative, as we have not considered wider benefits, for instance reducing what can be substantial mental and physical health impacts, as well as time out of work, to informal carers [76] of better AN treatment. There will be additional benefits if productivity losses related to lower rates of participation in employment by people with AN, as well as potentially reduced performance (presenteeism) while at work, can be reduced. These gains could be substantial. Health insurance claim data in Germany indicate employees with AN have an average of 73 days absenteeism in the year after diagnosis [77].

The model also does not directly capture potential reductions in mortality; a recent meta-analysis reported a mortality rate of 0.7% at 7-year follow-up from observational data, with longer waiting times associated with higher mortality [4]. Our measure of outcome, DALYs averted, is though weighted to take account of years of life lost due to AN, as well as years of life lived with AN.

Another challenge is that when using the DALY, the same disability weight is applied to all time spent living with AN. Therefore, our model assumes that individuals continue to experience the same level of AN disease burden regardless of differences in complexity or disease severity. While we mitigated this limitation by varying the disability weight attached to AN between 95% confidence intervals reported in the Global Burden of Disease study [78], and also varying assumptions on disability weight during periods of remission and recovery, future research might look at measures of quality of life associated with AN as an alternative. However, evidence on differences in utility weights used in estimating quality of life based on severity and/or complexity remain limited [79].

In the English model, our estimate of wait is based on data from a mixed ED population [55]. Ideally future analyses should use AN-specific wait times, as these are likely to be lower because of the severity of the condition. However, another English study, where 52% of the study population had AN, also reported a 27-week waiting time, conservatively only covering the period from referral, rather than first primary care visit [56].

We recognise our model provides a limited number of enhanced care pathway scenarios; future modelling work could consider additional further scenarios and population groups. For instance, although 78% of AN disease burden in Europe is in people aged over 20, the value of investing in enhanced care pathways for AN in adolescents also needs to be examined. Very low levels of transition from child and adolescent to adult ED services have been reported [80]; the majority of young adults might instead transition to generic services or be treated in primary care; both can lack appropriate training and skills [81].Yet, long-term impacts of AN emerging in adolescence are profound. In a 30-year follow-up study, they spent on average 10 years coping with AN; nearly 40% had another psychiatric disorder such as depression further impacting on cost [82]. Emerging US evidence indicates childhood AN, which is increasing in prevalence, may be associated with even worse long-term outcomes [83].

We have not considered differences in the value of care pathways by gender of care recipient. Although overall economic costs are similar, German analysis indicates rates of contact with outpatient services are lower for men; potentially this could reflect barriers in service access [84]. In England and Germany, we have assumed all inpatient care requires a stay in hospital, but some treatment may be offered by day care or home-treatment teams, but evidence on their effectiveness is still limited. Our model also assumes that specialist care is accessed via primary care but in all countries some individuals will be referred from acute care settings. Moreover, while primary care is the most common pathway in Germany, many adults access care via direct contact with specialists, including internal medicine, as well as psychiatry and psychotherapy [57]; care can also be provided exclusively on an inpatient basis [85].

Notwithstanding these limitations and future areas for research, our model suggests policy and practice guidelines should put an emphasis on enhanced care pathway measures to reduce wait times and enhance access to specialist care, as these have the potential both to improve outcomes and avert healthcare costs.

## Supporting information

McDaid et al. supplementary material McDaid et al. supplementary material

## Supplementary material

The supplementary material for this article can be found at http://doi.org/10.1192/j.eurpsy.2024.1751.
