# Supplementary material for: Quantifying the economic value of earlier and enhanced management of anorexia nervosa for adults in England, Germany and Spain: improving the care pathway
Source: Eur Psychiatry. 2024 May 23;67(1):e43. doi: 10.1192/j.eurpsy.2024.1751 (PMC11441344; doi:10.1192/j.eurpsy.2024.1751)
Supplement: McDaid et al. supplementary material [file S0924933824017516sup001.docx]

**Supplement 1: Sensitivity analysis**

Parameter uncertainty was addressed in multi-way sensitivity analyses**.** First one-way sensitivity analyses were conducted to look at how changes in model assumptions impact on expected costs of care pathways, NMB and relative ranking of care pathway scenarios. We varied model parameters by 20% above and below their baseline values. In addition, we varied the DALY disability weight for anorexia nervosa by their 95% confidence intervals (0.150 – 0.312). Further we varied our assumption that recovery and remission would have no adverse impacts at all and looked at how this would change if there was still some adverse DALY impact. We assumed would at maximum be the 0.150 DALY weight reported in the 95% confidence intervals. Our scenario 2 already indicated that the model is sensitive to changes in duration of expected wait time prior to access to specialist outpatient services; the longer the wait time the greater the economic case for taking steps to improve access to services, however in sensitivity none of the other parameters, including changes in the length of hospitalisation, use of specialists versus non-specialist outpatient care services or changes in the unit costs of care had a substantial impact on model results. Changes in DALY weights had no impact on overall ranking of model scenarios. The impacts of varying discount rates between 0% and 6% were also modest.

In addition, we undertook probabilistic sensitivity analysis varying key parameters concurrently 10,000 times, accounting for distributional assumptions around each parameter. Again, this did not change scenario rankings or impact substantially on the magnitude of NMBs.

**One-way sensitivity analyses**

**England Expected Mean Cost Lower Parameter Values**

| **Parameter** | **Scenario 1** | **% change** | **Scenario 2** | **% change** | **Scenario 3** | **% change** | **Scenario 4** | **% change** | **Scenario 5** | **% change** |
| --- | --- | --- | --- | --- | --- | --- | --- | --- | --- | --- |
| Unit cost ASED* inpatient (per day) | € 26,697 | -15.90% | € 22,890 | -15.64% | € 21,353 | -14.40% | € 19,373 | -14.24% | € 12,797 | -11.10% |
| Unit cost ASED* outpatient (Per contact) | € 30,951 | -2.50% | € 26,332 | -2.96% | € 23,809 | -4.55% | € 21,796 | -3.51% | € 13,249 | -7.96% |
| Unit Cost non-specialist outpatient (per contact) | € 31,502 | -0.77% | € 26,888 | -0.91% | € 24,944 | 0.00% | € 22,347 | -1.08% | € 14,395 | 0.00% |
| Unit cost Primary care doctor consultation (per contact) | € 31,482 | -0.83% | € 27,000 | -0.49% | € 24,681 | -1.05% | € 22,327 | -1.16% | € 14,261 | -0.93% |
| Length of inpatient stay | € 27,323 | -13.93% | € 23,412 | -13.72% | € 21,798 | -12.61% | € 19,769 | -12.49% | € 12,993 | -9.74% |
| Probability treated with non-specialist outpatient care | € 30,385 | -4.28% | € 26,007 | -4.15% | € 24,944 | 0.00% | € 21,052 | -6.81% | € 14,395 | 0.00% |
| Probability of hospitalisation following ASED outpatient treatment | € 29,232 | -7.92% | € 25,021 | -7.79% | € 21,353 | -14.40% | € 22,590 | 0.00% | € 14,395 | 0.00% |
| Probability of hospitalisation following non-specialist outpatient treatment | € 29,211 | -7.98% | € 25,003 | -7.85% | € 24,944 | 0.00% | € 20,481 | -9.34% | € 14,395 | 0.00% |
| Probability of rehospitalisation | € 30,304 | -4.54% | € 27,042 | -0.34% | € 23,919 | -4.11% | € 22,521 | -0.31% | € 14,361 | -0.24% |

- ASED=Adult Specialist Eating Disorder Service

**England Net Monetary Benefit Lower Parameter Values**

| **Parameter** | **Scenario 1** | **% change** | **Scenario 2** | **% change** | **Scenario 3** | **% change** | **Scenario 4** | **% change** | **Scenario 5** | **% change** |
| --- | --- | --- | --- | --- | --- | --- | --- | --- | --- | --- |
| DALY weight Anorexia Nervosa | € 231,273 | 1.77% | € 238,143 | 1.23% | € 238,294 | 1.67% | € 240,703 | 1.64% | € 251,279 | 1.09% |
| Discount rate (0%) | € 253,320 | 11.47% | € 262,211 | 11.46% | € 261,132 | 11.41% | € 263,842 | 11.41% | € 276,583 | 11.27% |

**England Expected Mean Cost Higher Parameter Values**

| **Parameter** | **Scenario 1** | **% change** | **Scenario 2** | **% change** | **Scenario 3** | **% change** | **Scenario 4** | **% change** | **Scenario 5** | **% change** |
| --- | --- | --- | --- | --- | --- | --- | --- | --- | --- | --- |
| Unit cost ASED* inpatient (per day) | € 36,793 | 15.90% | € 31,378 | 15.64% | € 28,534 | 14.39% | € 25,807 | 14.24% | € 15,993 | 11.10% |
| Unit cost ASED* outpatient (Per contact) | € 32,540 | 2.50% | € 27,937 | 2.96% | € 26,079 | 4.55% | € 23,385 | 3.52% | € 15,542 | 7.97% |
| Unit Cost non-specialist outpatient (per contact) | € 31,989 | 0.77% | € 27,381 | 0.91% | € 24,944 | 0.00% | € 22,834 | 1.08% | € 14,395 | 0.00% |
| Unit cost Primary care doctor consultation (per contact) | € 32,008 | 0.83% | € 27,269 | 0.50% | € 25,207 | 1.05% | € 22,853 | 1.16% | € 14,530 | 0.94% |
| Length of inpatient stay | € 36,153 | 13.89% | € 30,487 | 12.36% | € 28,079 | 12.57% | € 25,405 | 12.46% | € 15,793 | 9.71% |
| Probability treated with non-specialist outpatient care | € 33,106 | 4.29% | € 28,261 | 4.15% | € 24,944 | 0.00% | € 24,128 | 6.81% | € 14,395 | 0.00% |
| Probability of hospitalisation following ASED outpatient treatment | € 34,259 | 7.92% | € 29,247 | 7.79% | € 28,534 | 14.39% | € 22,590 | 0.00% | € 14,395 | 0.00% |
| Probability of hospitalisation following non-specialist outpatient treatment | € 34,280 | 7.99% | € 29,265 | 7.85% | € 24,944 | 0.00% | € 24,700 | 9.34% | € 14,395 | 0.00% |
| Probability of rehospitalisation | € 33,186 | 4.54% | € 28,498 | 5.03% | € 25,969 | 4.11% | € 23,624 | 4.58% | € 14,909 | 3.57% |

- ASED=Adult Specialist Eating Disorder Service

**England Net Monetary Benefit Higher Parameter Values**

| **Parameter** | **Scenario 1** | **% change** | **Scenario 2** | **% change** | **Scenario 3** | **% change** | **Scenario 4** | **% change** | **Scenario 5** | **% change** |
| --- | --- | --- | --- | --- | --- | --- | --- | --- | --- | --- |
| DALY weight Anorexia Nervosa | € 222,485 | -2.10% | € 231,794 | -1.47% | € 229,741 | -1.98% | € 232,211 | -1.95% | € 245,360 | -1.29% |
| DALY weight Remission / Recovery | € 194,723 | -14.32% | € 200,448 | -14.79% | € 201,632 | -13.97% | € 204,013 | -13.85% | € 213,383 | -14.16% |
| Discount rate (6%) | € 211,210 | -7.06% | € 218,622 | -7.07% | € 217,901 | -7.03% | € 220,167 | -7.03% | € 231,282 | -6.96% |

**Germany Expected Mean Cost Lower Parameter Values**

| **Parameter** | **Scenario 1** | **% change** | **Scenario 2** | **% change** | **Scenario 3** | **% change** | **Scenario 4** | **% change** | **Scenario 5** | **% change** |
| --- | --- | --- | --- | --- | --- | --- | --- | --- | --- | --- |
| Unit cost ASED* inpatient (per day) | € 11,191 | -17.23% | € 9,631 | -17.15% | € 8,784 | -15.88% | € 7,810 | -15.98% | € 4,947 | -13.18% |
| Unit cost ASED* outpatient (Per contact) | € 13,267 | -1.88% | € 11,365 | -2.24% | € 10,078 | -3.49% | € 9,041 | -2.73% | € 5,236 | -8.11% |
| Unit Cost non-specialist outpatient (per contact) | € 13,469 | -0.38% | € 11,572 | -0.46% | € 10,442 | 0.00% | € 9,243 | -0.56% | € 5,698 | 0.00% |
| Unit cost Primary care doctor consultation (per contact) | € 13,454 | -0.50% | € 11,609 | -0.14% | € 10,375 | -0.64% | € 9,228 | -0.72% | € 5,682 | -0.28% |
| Length of inpatient stay | € 11,868 | -12.23% | € 10,207 | -12.20% | € 9,265 | -11.27% | € 8,239 | -11.36% | € 5,164 | -9.37% |
| Probability treated with non-specialist outpatient care | € 12,905 | -4.56% | € 11,108 | -4.45% | € 10,442 | 0.00% | € 8,597 | -7.51% | € 5,698 | 0.00% |
| Probability of hospitalisation following ASED outpatient treatment | € 12,361 | -8.58% | € 10,632 | -8.54% | € 9,295 | -10.98% | € 8,784 | -5.50% | € 5,698 | 0.00% |
| Probability of hospitalisation following non-specialist outpatient treatment | € 12,351 | -8.65% | € 10,624 | -8.61% | € 10,442 | 0.00% | € 8,321 | -10.48% | € 5,698 | 0.00% |
| Probability of rehospitalisation | € 12,856 | -4.92% | € 11,582 | -0.37% | € 9,969 | -4.53% | € 9,263 | -0.34% | € 5,682 | -0.28% |

- ASED=Adult Specialist Eating Disorder Service

**Germany Net Monetary Benefit Lower Parameter Values**

| **Parameter** | **Scenario 1** | **% change** | **Scenario 2** | **% change** | **Scenario 3** | **% change** | **Scenario 4** | **% change** | **Scenario 5** | **% change** |
| --- | --- | --- | --- | --- | --- | --- | --- | --- | --- | --- |
| DALY weight Anorexia Nervosa | €251,978 | 1.12% | €255,485 | 0.79% | €255,236 | 1.07% | €256,429 | 1.06% | €261,742 | 0.71% |
| Discount rate (0%) | €276,842 | 11.10% | €281,567 | 11.08% | €280,451 | 11.06% | €281,773 | 11.04% | €288,427 | 10.97% |

**Germany Expected Mean Cost Higher Parameter Values**

| **Parameter** | **Scenario 1** | **% change** | **Scenario 2** | **% change** | **Scenario 3** | **% change** | **Scenario 4** | **% change** | **Scenario 5** | **% change** |
| --- | --- | --- | --- | --- | --- | --- | --- | --- | --- | --- |
| Unit cost ASED* inpatient (per day) | € 15,851 | 17.23% | € 13,620 | 17.16% | € 12,099 | 15.87% | € 10,780 | 15.98% | € 6,449 | 13.18% |
| Unit cost ASED* outpatient (Per contact) | € 13,775 | 1.88% | € 11,886 | 2.25% | € 10,805 | 3.48% | € 9,549 | 2.73% | € 6,070 | 6.53% |
| Unit Cost non-specialist outpatient (per contact) | € 13,573 | 0.38% | € 11,678 | 0.46% | € 10,442 | 0.00% | € 9,347 | 0.56% | € 5,698 | 0.00% |
| Unit cost Primary care doctor consultation (per contact) | € 13,588 | 0.50% | € 11,642 | 0.15% | € 10,509 | 0.64% | € 9,362 | 0.72% | € 5,714 | 0.28% |
| Length of inpatient stay | € 15,995 | 18.30% | € 13,748 | 18.26% | € 12,201 | 16.85% | € 10,874 | 16.99% | € 6,497 | 14.02% |
| Probability treated with non-specialist outpatient care | € 14,137 | 4.56% | € 12,143 | 4.46% | € 10,442 | 0.00% | € 9,993 | 7.51% | € 5,698 | 0.00% |
| Probability of hospitalisation following ASED outpatient treatment | € 14,682 | 8.59% | € 12,619 | 8.55% | € 12,100 | 15.88% | € 9,295 | 0.00% | € 5,698 | 0.00% |
| Probability of hospitalisation following non-specialist outpatient treatment | € 14,692 | 8.66% | € 12,627 | 8.62% | € 10,442 | 0.00% | € 10,269 | 10.48% | € 5,698 | 0.00% |
| Probability of rehospitalisation | € 14,186 | 4.92% | € 12,268 | 5.53% | € 10,915 | 4.53% | € 9,772 | 5.13% | € 5,940 | 4.25% |

- ASED=Adult Specialist Eating Disorder Service

**Germany Net Monetary Benefit Higher Parameter Values**

| **Parameter** | **Scenario 1** | **% change** | **Scenario 2** | **% change** | **Scenario 3** | **% change** | **Scenario 4** | **% change** | **Scenario 5** | **% change** |
| --- | --- | --- | --- | --- | --- | --- | --- | --- | --- | --- |
| DALY weight Anorexia Nervosa | € 245,868 | -1.33% | € 251,115 | -0.94% | € 249,319 | -1.27% | € 250,561 | -1.26% | € 257,730 | -0.84% |
| DALY weight Remission / Recovery | € 214,170 | -14.05% | € 216,861 | -14.45% | € 217,338 | -13.94% | € 218,508 | -13.89% | € 222,952 | -14.22% |
| Discount rate (6%) | € 232,113 | -6.85% | € 236,148 | -6.84% | € 235,189 | -6.87% | € 236,436 | -6.82% | € 242,282 | -6.78% |

**Spain Expected Mean Cost Lower Parameter Values**

| **Parameter** | **Scenario 1** | **% change** | **Scenario 2** | **% change** | **Scenario 3** | **% change** | **Scenario 4** | **% change** | **Scenario 5** | **% change** |
| --- | --- | --- | --- | --- | --- | --- | --- | --- | --- | --- |
| Unit cost ASED* inpatient (per day) | € 13,933 | -14.66% | € 12,229 | -14.07% | € 10,917 | -13.49% | € 10,028 | -13.22% | € 6,355 | -10.61% |
| Unit cost ASED* outpatient (Per contact) | € 15,974 | -2.16% | € 13,878 | -2.49% | € 12,116 | -3.99% | € 11,202 | -3.05% | € 6,603 | -7.12% |
| Unit Cost non-specialist outpatient (per contact) | € 16,233 | -0.57% | € 14,138 | -0.66% | € 12,619 | 0.00% | € 11,461 | -0.81% | € 7,109 | 0.00% |
| Unit cost Primary care doctor consultation (per contact) | € 16,275 | -0.31% | € 14,211 | -0.15% | € 12,568 | -0.40% | € 11,503 | -0.45% | € 7,088 | -0.30% |
| Unit cost day hospital contact (per day) | € 15,952 | -2.29% | € 13,856 | -2.64% | € 12,353 | -2.11% | € 11,268 | -2.48% | € 6,968 | -1.98% |
| Length of inpatient stay | € 14,344 | -12.14% | € 12,573 | -11.66% | € 11,209 | -11.17% | € 10,289 | -10.96% | € 6,484 | -8.79% |
| Length of day hospital treatment | € 15,984 | -2.09% | € 13,884 | -2.45% | € 12,376 | -1.93% | € 11,289 | -2.30% | € 6,978 | -1.84% |
| Probability treated with non-specialist outpatient care | € 15,585 | -4.54% | € 13,603 | -4.42% | € 12,619 | 0.00% | € 10,691 | -7.48% | € 7,109 | 0.00% |
| Probability of hospitalisation following ASED outpatient treatment | € 14,948 | -8.44% | € 13,048 | -8.32% | € 10,650 | -15.60% | € 11,555 | 0.00% | € 7,109 | 0.00% |
| Probability of hospitalisation following non-specialist outpatient treatment | € 14,937 | -8.51% | € 13,038 | -8.39% | € 12,619 | 0.00% | € 10,365 | -10.30% | € 7,109 | 0.00% |
| Probability of rehospitalisation | € 15,647 | -4.16% | € 14,189 | -0.30% | € 12,136 | -3.83% | € 11,522 | -0.29% | € 7,093 | -0.23% |

- ASED=Adult Specialist Eating Disorder Service

**Spain Net Monetary Benefit Lower Parameter Values**

| **Parameter** | **Scenario 1** | **% change** | **Scenario 2** | **% change** | **Scenario 3** | **% change** | **Scenario 4** | **% change** | **Scenario 5** | **% change** |
| --- | --- | --- | --- | --- | --- | --- | --- | --- | --- | --- |
| DALY weight Anorexia Nervosa | € 249,369 | 1.09% | € 252,383 | 0.90% | € 253,381 | 1.01% | € 254,458 | 1.01% | € 260,109 | 0.75% |
| Discount rate (0%) | € 274,240 | 11.17% | € 278,019 | 11.14% | € 278,739 | 11.12% | € 279,935 | 11.12% | € 286,603 | 11.01% |

**Spain Expected Mean Cost Higher Parameter Values**

| **Parameter** | **Scenario 1** | **% change** | **Scenario 2** | **% change** | **Scenario 3** | **% change** | **Scenario 4** | **% change** | **Scenario 5** | **% change** |
| --- | --- | --- | --- | --- | --- | --- | --- | --- | --- | --- |
| Unit cost ASED* inpatient (per day) | € 18,720 | 14.66% | € 16,234 | 14.07% | € 14,322 | 13.50% | € 13,082 | 13.22% | € 7,863 | 10.61% |
| Unit cost ASED* outpatient (Per contact) | € 16,679 | 2.16% | € 14,586 | 2.49% | € 13,123 | 3.99% | € 11,907 | 3.05% | € 7,615 | 7.12% |
| Unit Cost non-specialist outpatient (per contact) | € 16,420 | 0.58% | € 14,325 | 0.65% | € 12,619 | 0.00% | € 11,648 | 0.80% | € 7,109 | 0.00% |
| Unit cost Primary care doctor consultation (per contact) | € 16,378 | 0.32% | € 14,252 | 0.14% | € 12,670 | 0.40% | € 11,606 | 0.44% | € 7,130 | 0.30% |
| Unit cost day hospital contact (per day) | € 16,701 | 2.30% | € 14,607 | 2.63% | € 12,885 | 2.11% | € 11,841 | 2.48% | € 7,250 | 1.98% |
| Length of inpatient stay | € 18,304 | 12.12% | € 15,888 | 11.64% | € 14,026 | 11.15% | € 12,818 | 10.93% | € 7,733 | 8.78% |
| Length of day hospital treatment | € 16,688 | 2.22% | € 14,579 | 2.44% | € 12,862 | 1.93% | € 11,820 | 2.29% | € 7,240 | 1.84% |
| Probability treated with non-specialist outpatient care | € 17,068 | 4.54% | € 14,860 | 4.41% | € 12,619 | 0.00% | € 12,419 | 7.48% | € 7,109 | 0.00% |
| Probability of hospitalisation following ASED outpatient treatment | € 17,705 | 8.45% | € 15,416 | 8.32% | € 14,588 | 15.60% | € 11,555 | 0.00% | € 7,109 | 0.00% |
| Probability of hospitalisation following non-specialist outpatient treatment | € 17,716 | 8.51% | € 15,426 | 8.39% | € 12,619 | 0.00% | € 12,744 | 10.29% | € 7,109 | 0.00% |
| Probability of rehospitalisation | € 17,006 | 4.17% | € 14,871 | 4.49% | € 13,103 | 3.84% | € 12,042 | 4.21% | € 7,350 | 3.39% |

- ASED=Adult Specialist Eating Disorder Service

**Spain Net Monetary Benefit Higher Parameter Values**

| **Parameter** | **Scenario 1** | **% change** | **Scenario 2** | **% change** | **Scenario 3** | **% change** | **Scenario 4** | **% change** | **Scenario 5** | **% change** |
| --- | --- | --- | --- | --- | --- | --- | --- | --- | --- | --- |
| DALY weight Anorexia Nervosa | € 243,472 | -1.30% | € 247,478 | -1.06% | € 247,814 | -1.21% | € 248,903 | -1.20% | € 255,857 | -0.89% |
| DALY weight Remission / Recovery | € 211,462 | -14.28% | € 214,010 | -14.44% | € 215,320 | -14.16% | € 216,390 | -14.10% | €221,431 | -14.23% |
| Discount rate (6%) | € 229,665 | -6.90% | € 232,932 | -6.88% | € 233,609 | -6.87% | € 234,620 | -6.87% | € 240,593 | -6.81% |

**Probabilistic Sensitivity Analysis Results**

**England**

**
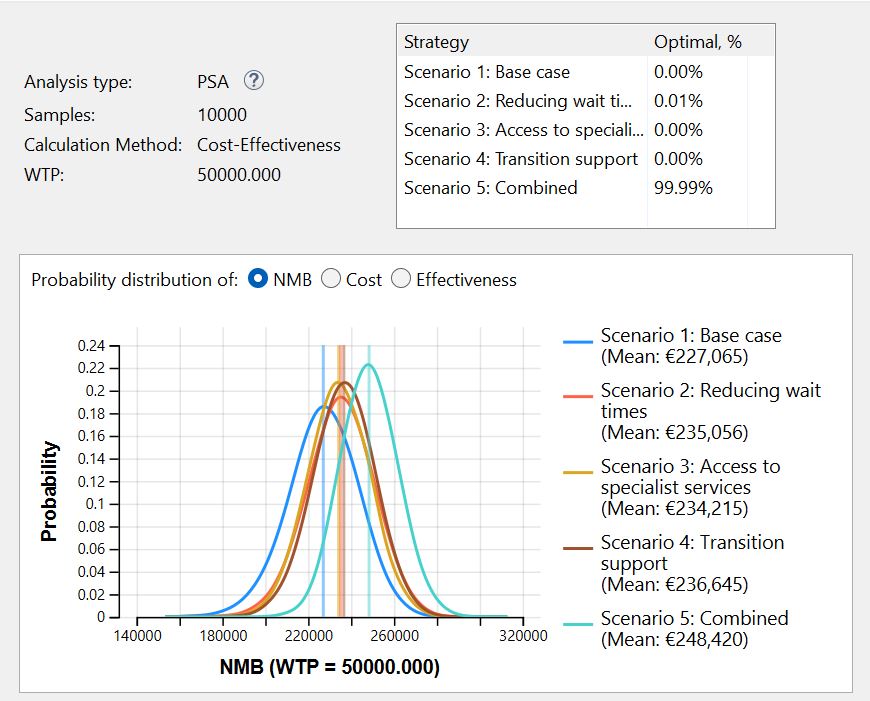
**

**Germany**

**
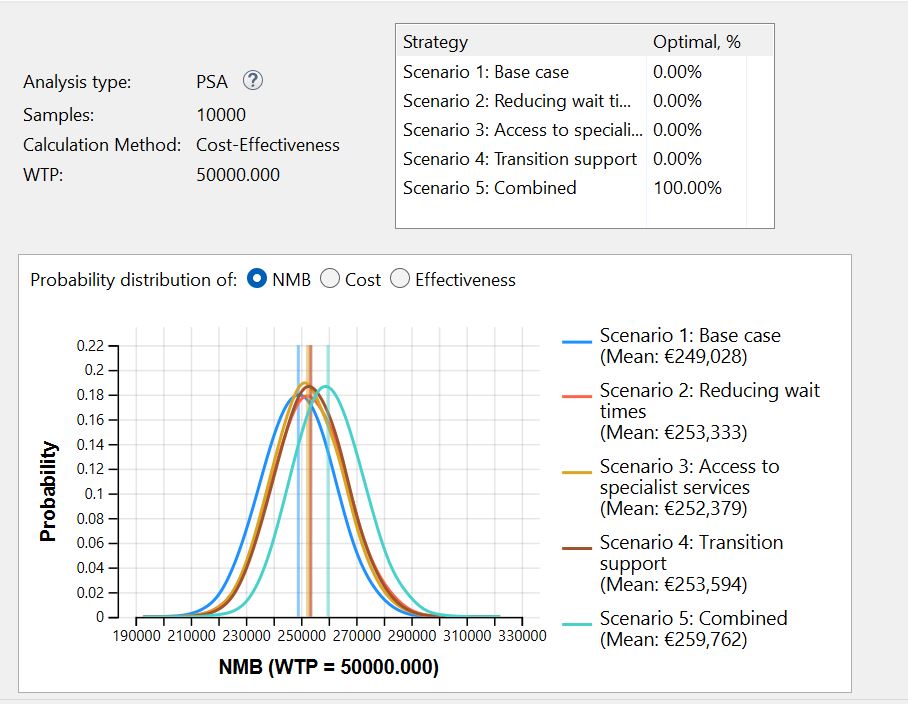
**

**Spain**

**
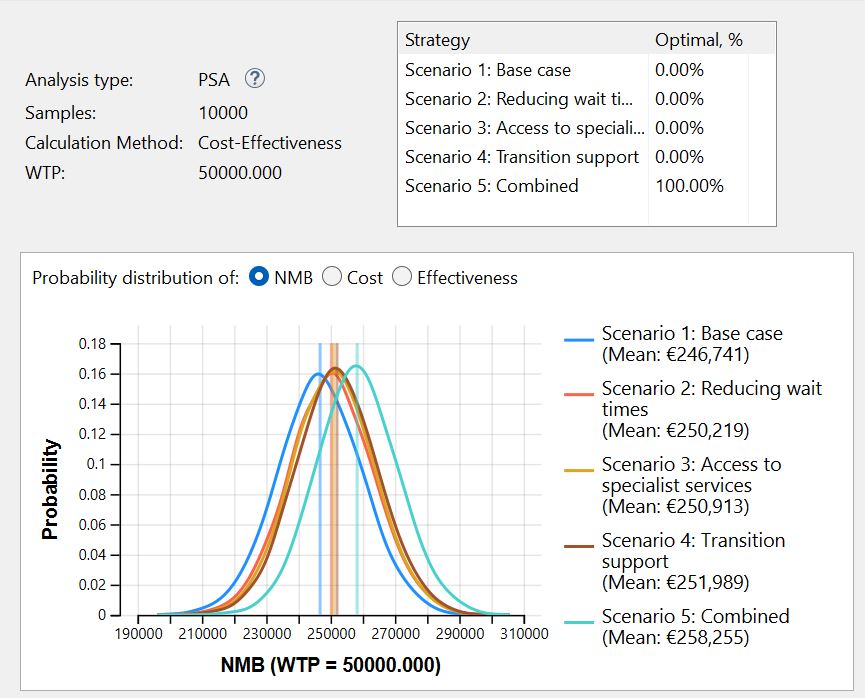
**

**Supplement 2: Consolidated Health Economic Evaluation Reporting Standards (CHEERS) statement**

# CHEERS 2022 Checklist

| **Topic** | **No.** | **Item** | **Location where item is reported** |
| --- | --- | --- | --- |
| **Title** |  |  |  |
|  | 1 | Identify the study as an economic evaluation and specify the interventions being compared. | Title, Line1, Page 3 |
| **Abstract** |  |  |  |
|  | 2 | Provide a structured summary that highlights context, key methods, results, and alternative analyses. | Abstract, Page 3, Lines 4-23, Page 3, Lines 24-32 |
| **Introduction** |  |  |  |
| **Background and objectives** | 3 | Give the context for the study, the study question, and its practical relevance for decision making in policy or practice. | Introduction, Page 5, Lines 39 to Page 7, Line 127. |
| **Methods** |  |  |  |
| **Health economic analysis plan** | 4 | Indicate whether a health economic analysis plan was developed and where available. | N/A |
| **Study population** | 5 | Describe characteristics of the study population (such as age range, demographics, socioeconomic, or clinical characteristics). | Page 9, Line 136-137 |
| **Setting and location** | 6 | Provide relevant contextual information that may influence findings. | Page 6, Line 70 – Page 8, Line 112, Figure 2 |
| **Comparators** | 7 | Describe the interventions or strategies being compared and why chosen. | Care Pathway Scenarios, Page 9, Line 145 – Page 10, Line 173 |
| **Perspective** | 8 | State the perspective(s) adopted by the study and why chosen. | Page 11, Line 184-185. |
| **Time horizon** | 9 | State the time horizon for the study and why appropriate. | Page 8, Line 138-139. |
| **Discount rate** | 10 | Report the discount rate(s) and reason chosen. | Page 10, Line 183, Page 11 Line 184. |
| **Selection of outcomes** | 11 | Describe what outcomes were used as the measure(s) of benefit(s) and harm(s). | Page 10, Lines 174-179 and Page 11, Lines 186-191. |
| **Measurement of outcomes** | 12 | Describe how outcomes used to capture benefit(s) and harm(s) were measured. | Page 10, Lines 174-179 and Page 11, Lines 186-191. |
| **Valuation of outcomes** | 13 | Describe the population and methods used to measure and value outcomes. | Page 10, Lines 174-179 and Page 11, Lines 186-191. |
| **Measurement and valuation of resources and costs** | 14 | Describe how costs were valued. | Table 1 |
| **Currency, price date, and conversion** | 15 | Report the dates of the estimated resource quantities and unit costs, plus the currency and year of conversion. | Page 10, Lines 180-183, and Table 1 |
| **Rationale and description of model** | 16 | If modelling is used, describe in detail and why used. Report if the model is publicly available and where it can be accessed. | Page 8, Lines 130 – 132, Page 9 Lines 133 – 156, Page 10 Lines 157-180, Page 11, Lines 181-204, Page 12 Lines 205 – 208. Figure 1, Data availability statement |
| **Analytics and assumptions** | 17 | Describe any methods for analysing or statistically transforming data, any extrapolation methods, and approaches for validating any model used. | N/A |
| **Characterising heterogeneity** | 18 | Describe any methods used for estimating how the results of the study vary for subgroups. | Care Pathway Scenarios, Page 8, Line 145 – Page 10, Line 173 |
| **Characterising distributional effects** | 19 | Describe how impacts are distributed across different individuals or adjustments made to reflect priority populations. | Page 11, Line 196 – Page 12 Line 205, for country-specific results |
| **Characterising uncertainty** | 20 | Describe methods to characterise any sources of uncertainty in the analysis. | Page 13, Lines 248 – Page 14, 262 |
| **Approach to engagement with patients and others affected by the study** | 21 | Describe any approaches to engage patients or service recipients, the general public, communities, or stakeholders (such as clinicians or payers) in the design of the study. | N/A |
| **Results** |  |  |  |
| **Study parameters** | 22 | Report all analytic inputs (such as values, ranges, references) including uncertainty or distributional assumptions. | Table 1 |
| **Summary of main results** | 23 | Report the mean values for the main categories of costs and outcomes of interest and summarise them in the most appropriate overall measure. | Results, Page 12, Lines 226-232 and Page 13, Lines 233-247, Tables 2, 3, and 4, Figure 3 and Figure 4 |
| **Effect of uncertainty** | 24 | Describe how uncertainty about analytic judgments, inputs, or projections affect findings. Report the effect of choice of discount rate and time horizon, if applicable. | Page 13, Lines 248 – Page 14, 262, Supplement |
| **Effect of engagement with patients and others affected by the study** | 25 | Report on any difference patient/service recipient, general public, community, or stakeholder involvement made to the approach or findings of the study | N/A |
| **Discussion** |  |  |  |
| **Study findings, limitations, generalisability, and current knowledge** | 26 | Report key findings, limitations, ethical or equity considerations not captured, and how these could affect patients, policy, or practice. | Discussion, From Page 14, Line 263 to Page 18, Line 377. |
| **Other relevant information** |  |  |  |
| **Source of funding** | 27 | Describe how the study was funded and any role of the funder in the identification, design, conduct, and reporting of the analysis | Financial Support, Page 19, Line 378 - 390 |
| **Conflicts of interest** | 28 | Report authors conflicts of interest according to journal or International Committee of Medical Journal Editors requirements. | Conflicts of Interest, Page 19, From Line 391 |
